# *In Vivo* Efficacy of the Combination of Ciprofloxacin and Cefotaxime against *Vibrio vulnificus* Sepsis

**DOI:** 10.1371/journal.pone.0101118

**Published:** 2014-06-30

**Authors:** Hee-Chang Jang, Su-Mi Choi, Hee Kyung Kim, Sung-Eun Kim, Seung-Ji Kang, Kyung-Hwa Park, Phil Youl Ryu, Tae-Hoon Lee, Young Ran Kim, Joon Haeng Rhee, Sook-In Jung, Hyon E Choy

**Affiliations:** 1 Department of Infectious diseases, Chonnam National University Medical School, Gwang-ju, Republic of Korea; 2 Research Institute of Vibrio Infection and Genome Research Center for Enteropathogenic Bacteria, Gwang-ju, Republic of Korea; 3 Microbiology, Chonnam National University Medical School, Gwang-ju, Republic of Korea; 4 Department of Biochemistry, School of Dentistry, Chonnam National University, Gwang-ju, Republic of Korea; 5 Department of Pharmaceutical Engineering, Dongshin University, Naju, Republic of Korea; Technische Universität Dresden, Medical Faculty, Germany

## Abstract

**Objectives:**

The *in* *vivo* efficacy of a cefotaxime-ciprofloxacin combination against *Vibrio vulnificus* and the effects on *rtxA1* expression of commonly used antibiotics are unknown.

**Methods:**

*In vitro* time-kill studies were performed to evaluate synergism. Female BALB/c mice were injected subcutaneously with 1×10^7^ or 1×10^8^ cfu of *V. vulnificus*. Antibiotic therapy was initiated at 2 h after inoculation in the following four therapy groups: cefotaxime; ciprofloxacin; cefotaxime-plus-ciprofloxacin; and cefotaxime-plus-minocycline. The cytotoxicity of *V. vulnificus* for HeLa cells was measured using the lactate dehydrogenase assay; *rtxA1* transcription was measured in a transcriptional reporter strain using a β-galactosidase assay.

**Results:**

*In vitro* time-kill assays exhibited synergism between cefotaxime and ciprofloxacin. In the animal experiments, the 96-h survival rate for the cefotaxime-plus-ciprofloxacin group (85%; 17/20) was significantly higher than that of the cefotaxime-plus-minocycline (35%; 7/20) and cefotaxime alone (0%; 0/20) groups (*P*<0.05 for both). Bacterial counts in the liver and spleen were significantly lower in the cefotaxime-plus-ciprofloxacin group 24 and 48 h after treatment, relative to the other groups. At sub-inhibitory concentrations, ciprofloxacin inhibited more effectively *rtxA1* transcription and mammalian cell cytotoxicity than either minocycline or cefotaxime (*P*<0.05 for both).

**Conclusions:**

Ciprofloxacin is more effective at reducing *rtxA1* transcription and subsequent cytotoxicity than either minocycline or cefotaxime, and the combination of ciprofloxacin and cefotaxime was more effective in clearing *V. vulnificus in* *vivo* than previously used regimens. These data suggest that the combination of ciprofloxacin and cefotaxime is an effective option for the treatment of *V. vulnificus* sepsis in humans.

## Introduction

*Vibrio vulnificus* is an opportunistic human pathogen that causes rapidly fatal sepsis, as well as skin and soft tissue infections, including necrotizing fasciitis. This pathogen is transmitted to humans through the exposure of wounds to seawater or the ingestion of seafood, and causes septicemia, especially in patients with immunosuppression and iron overload, as is the case in chronic liver disease [1], [2]. The mortality rate for *V. vulnificus* septicemia is ∼70% [1], and the major virulence factors of this pathogen include capsular polysaccharides, exotoxins such as the repeats-in-toxin A1 (RtxA1), hemolysin, metalloproteinase, and iron acquisition systems. Among these, RtxA1 is a potent cytotoxic virulence factor that plays important roles in the pathogenesis and lethality of *V. vulnificus* infections [3]–[10].

Immediate surgical intervention and the administration of effective antibiotics are essential to reduce the mortality rate for *V. vulnificus* sepsis [1], [2], [11]. Antibiotics that are known to be effective include beta-lactams, tetracyclines, and quinolones. Among these, the combinations of a third-generation cephalosporin with tetracycline or quinolone monotherapy are most often recommended, based on the results of *in* *vitro* [12] and *in* *vivo* research [13], [14] and several retrospective clinical studies [15], [16]. However, the mortality rates remain high, suggesting the need for adjuvant therapies [17], [18] and more effective antibiotic regimens.

A combination of cefotaxime and a quinolone has a synergistic effect on enteric Gram-negative pathogens, including *Escherichia coli* [19] and *Salmonella* species [20], [21]. It has also been shown that the combination of cefotaxime and ciprofloxacin has synergism and superior *in* *vitro* bactericidal activity against *V. vulnificus* than currently recommended regimens, such as ciprofloxacin alone or cefotaxime plus doxycycline [22]. However, the *in* *vivo* efficacy of this combination remains unclear. Moreover, although RtxA1 plays a key role in the virulence of *V. vulnificus* and mortality in *V. vulnificus*-related sepsis, the effects of commonly used therapeutic antibiotics at sub-inhibitory concentrations on *rtxA1* expression have not been evaluated. To address these issues, we evaluated the *in* *vitro* synergism and *in* *vivo* efficacy of the combination of ciprofloxacin and cefotaxime against *V. vulnificus* sepsis, as well as the effect of commonly used antibiotics on *rtxA1* expression.

## Materials and Methods

### Ethics

All animal experiments were carried out in accordance with the guidelines set forth by the Institutional Animal Care and Use Committee (IACUC) of Chonnam National University and the guidelines for animal experiments set forth by the Korean Food and Drug Administration (KFDA) [23]. The study protocol was approved by the IACUC of Chonnam National University Hwasun Hospital.

### Bacterial strains and *in* *vitro* time-kill assay

*V. vulnificus* CMCP6, which is a clinical isolate from Chonnam National University Hospital for which full genome sequence is available (GenBank accession nos. AEO16795 and AEO16796) [24], was used in the time-kill study and animal infection study. *V. vulnificus* strains MO6-24/O and CMM770 (MO6-24/O background with a deletion mutation in the *rtxA1* gene) [25] were used in the cytotoxicity assay and transcriptional reporter assay.

The minimal inhibitory concentrations (MICs) of cefotaxime, minocycline, and ciprofloxacin were determined by the microdilution method according to the guidelines of the Clinical and Laboratory Standards Institute [26]. *In vitro* time-kill studies were performed to evaluate synergy, as described previously [22]. Cefotaxime (Chong Kun Dang Pharmaceutical, Seoul, Republic of Korea), minocycline (Sigma-Aldrich, St. Louis, MO) and ciprofloxacin (Ildong Pharmaceutical, Seoul, Republic of Korea) were used throughout the study. Synergy was defined as a ≥2–log_10_ cfu/mL increase in killing at 24 h using the combination therapy compared with the level of killing achieved with the most active single drug.

### *In vivo* animal study

Female, specific pathogen free, 8-week-old BALB/c mice (Samtako, Osan, Republic of Korea) with an average weight of 20 g were used throughout the study. The inocula were prepared as described previously [14]. We chose 1×10^7^ and 1×10^8^ cfu as the initial inocula, based on previous studies [12], [14] and our preliminary results. To induce an iron-overload status, which increases susceptibility to *V. vulnificus* and more closely represents the iron-overloaded condition seen clinically, 900 µg ferric ammonium citrate was administered intra-peritoneally (i.p.) 30 min before *V. vulnificus* inoculation [27]. Next, 1×10^7^ or 1×10^8^ cfu *V. vulnificus* were injected subcutaneously into the area over the right thigh [12], [14].

Each experiment consisted of five groups: a control group and groups treated with cefotaxime, ciprofloxacin, cefotaxime-plus-ciprofloxacin, and cefotaxime-plus-minocycline. All antibiotics were initially given i.p. beginning 2 h after the animal was infected. Cefotaxime (30 mg/kg body weight [BW] i.p.) was given every 6 h. Minocycline (loading dose of 4 mg/kg BW, followed by a maintenance dose of 2 mg/kg BW i.p.) was given every 12 h. Ciprofloxacin (8 mg/kg BW, i.p.) was given every 12 h, as described previously [12], [14]. Control mice received 0.1 mL sterile saline every 6 h. Antibiotics were given for a total of 42 h. The condition of animals was monitored every 6 h. Humane endpoints were used during the survival study; animals were euthanized using ether when they exhibited a combined clinical criteria (≥8 points), according to KFDA guidelines [23]. Clinical endpoints were defined using the following scoring system: change in body weight, 0–3 points; hair coat, 0–2 points; eye opening, 0–2 points; activity, 0–2 points; posture 0–3 points.

In addition, we counted the numbers of viable bacteria in the livers and spleens 24 and 48 h of infected mice after initiation of antibiotic treatment using an initial inoculum of 1×10^7^ cfu. Mice were humanely euthanized using ether at 24 and 48 h, and the livers and spleens were homogenized. Then, the homogenized tissues were serially diluted and plated onto Brain-Heart infusion agar, to quantify the bacteria.

### Effects of sub-inhibitory concentrations of antibiotics on *V. vulnificus* cytotoxicity for HeLa cells

*V. vulnificus* cytotoxicity for HeLa cells was measured using the CytoTox96 non-radioactive cytotoxicity assay kit (Promega, Madison, WI), as described elsewhere [28], [29]. HeLa cells were seeded in 48-well culture plates and cultured at 37°C in 5% CO_2_. After incubation for 24 h, the cells were washed twice with pre-warmed serum-free Dulbecco’s modified Eagle’s medium (DMEM). Overnight-cultured *V. vulnificus* bacteria were incubated in fresh 2.5% NaCl heart infusion (HI) broth for 3 h. The logarithmically growing culture was harvested by centrifugation, washed with PBS, and re-suspended in PBS. Then, 2×10^5^ HeLa cells/mL in 250 µL of DMEM were infected with 5×10^5^ cfu/mL *V. vulnificus* with or without 1/4 MICs of antibiotics for 120 min. Lactate dehydrogenase (LDH) released into the supernatant fluid was assayed as a marker of cytotoxicity, in accordance with the manufacturer’s protocol.

### Effects of sub-inhibitory concentrations of antibiotics on *rtxA1* expression

A chromosomal P*rtxA1::lacZ* transcriptional reporter strain of *V. vulnificus* MO6–24/O was constructed as described previously [29], [30]. An overnight culture of the reporter strain was inoculated in 2.5% NaCl HI broth at a concentration of 5×10^5^ cfu/mL with or without 1/4 MICs of antibiotics for 120 min. The culture was lysed in lysis buffer, and β-galactosidase activity was assayed using 2×β-galactosidase substrate (0.2 M sodium phosphate buffer, 2 mM MgCl_2_, 100 mM mercaptoethanol, 1.33 mg/mL O-nitrophenyl-β-D-galactopyranoside), following the method described previously [30]. All experiments were performed three or more times.

### Statistical analyses

The Kolmogorov–Smirnov goodness-of-fit test was used to determine the distribution of each set of data for normality before analysis, and continuous variables were compared using Student’s *t-*test. Survival analysis was performed using the Kaplan–Meier method and log-rank test. All tests of significance were two-tailed, and *P*-values≤0.05 were deemed to indicate statistical significance. Statistical analyses of the data were performed using the SPSS ver. 19.0 software (SPSS, Chicago, IL) and the GraphPad Prism ver. 5.0 program (GraphPad Software, La Jolla, CA).

## Results

### *In vitro* time-kill assay

**Figure 1:**
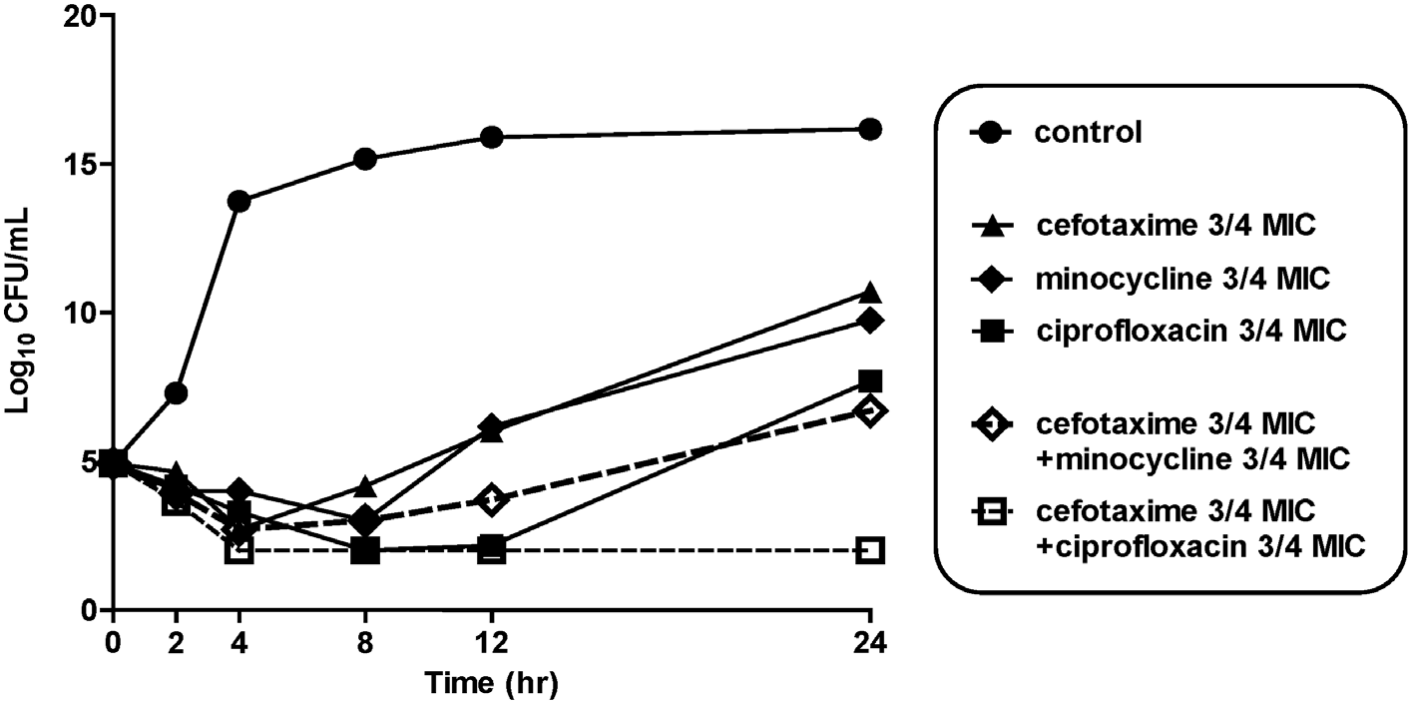
Time-kill curves for *V. vulnificus* CMCP6 after incubation with 3/4 MICs of cefotaxime alone, minocycline alone, ciprofloxacin alone, cefotaxime-plus-ciprofloxacin or cefotaxime-plus-minocycline. CFU, colony-forming unit; MIC, minimum inhibitory concentration.

The MICs of cefotaxime, minocycline, and ciprofloxacin for *V. vulnificus* CMCP6 were 0.0625, 0.0625, and 0.03 mg/L, respectively. In the time-kill assay using 3/4 MICs of each antibiotic, the cefotaxime-ciprofloxacin combination was found to have synergistic activity against *V. vulnificus* CMCP6, and the bacterial colony counts after 24 h of *in* *vitro* treatment were lower for cefotaxime-plus-ciprofloxacin than for cefotaxime, ciprofloxacin, minocycline or cefotaxime-plus-minocycline (Fig. 1).

### Survival rates of mice with *V. vulnificus* sepsis treated with various antibiotic regimens

**Figure 2:**
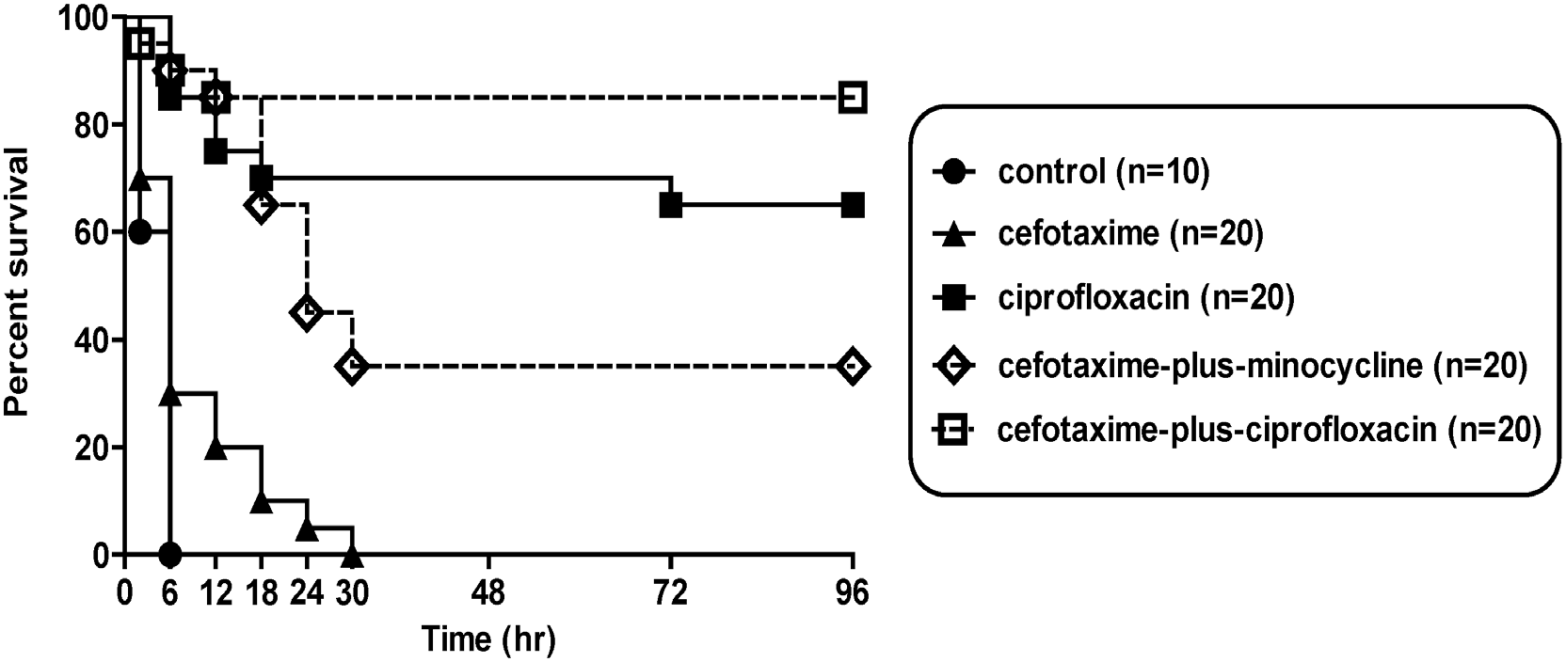
Survival rates of mice in each treatment group inoculated with 1×10^8^ cfu *V. vulnificus*. The 96-h survival rate of the cefotaxime-plus-ciprofloxacin group (85%, 17/20) was significantly higher than that of the cefotaxime (0%, 0/20) or the cefotaxime-plus-minocycline groups (35%, 7/20) (*P*<0.001 and *P = *0.003, respectively; log-rank test).

Figure 2 presents the survival rates of each treatment group after inoculation with 1×10^8^ cfu *V. vulnificus* CMCP6. All 10 control mice died within 12 h. The 96-h survival rate was significantly higher in the cefotaxime-plus-ciprofloxacin group (85%, 17/20) compared with the cefotaxime (0%, 0/20) and cefotaxime-plus-minocycline groups (35%, 7/20) (*P*<0.001, *P = *0.003, respectively). The 96-h survival rate in the cefotaxime-plus-ciprofloxacin group was also higher than that in the ciprofloxacin group (65%, 13/20), although the difference was not statistically significant (*P* = 0.148).

### *In vivo* clearance of *V. vulnificus* from mice treated with various antibiotic regimens

In experiments where 1×10^7^ cfu *V. vulnificus* CMCP6 was used as the initial inoculum, all infected control mice (n = 6) died within 12 h, whereas all 48 of the infected mice treated with cefotaxime (n = 12), ciprofloxacin (n = 12), cefotaxime-plus-ciprofloxacin (n = 12), or cefotaxime-plus-minocycline (n = 12) were still alive after 96 h.

The viable bacterial counts in liver were lower in mice treated with cefotaxime-plus-ciprofloxacin than in those treated with cefotaxime alone (*P*<0.001 at 24 h and 48 h, each), ciprofloxacin alone (*P* = 0.030 at 24 h; *P* = 0.001 at 48 h) and cefotaxime-plus-minocycline (*P* = 0.044 at 24 h; *P* = 0.008 at 48 h). The viable bacterial counts in spleen were lower in mice treated with cefotaxime-plus-ciprofloxacin than those treated with cefotaxime alone (*P*<0.001) and ciprofloxacin alone (*P* = 0.003) at 24 h and those treated with cefotaxime alone (*P*<0.001) and cefotaxime-plus-minocycline (*P* = 0.008) at 48 h (n = 9 mice per group; Table 1).

**Table 1:** Viable bacterial counts in the livers and spleens 24 and 48×10^7^ cfu *V. vulnificus* (n = 9 per group).

Bacterial counts (CFU/g)	Treatment group				*P* value^a^	*P* value^b^	*P* value^c^
	Cefotaxime	Ciprofloxacin	Cefotaxime-plus-minocycline	Cefotaxime-plus-ciprofloxacin			
Liver at 24 h	19544±12097	1415±1144	911±516	473±309	<0.001	0.030	0.044
Liver at 48 h	921±101	310±109	287±119	162±35	<0.001	0.001	0.008
Spleen at 24 h	13180±5558	3234±1092	2617±1413	1813±514	<0.001	0.003	0.128
Spleen at 48 h	1304±565	379±238	361±113	217±87	<0.001	0.074	0.008

Bacterial counts were expressed as means ± SDs.

CFU, colony-forming unit.

a Comparison of the cefotaxime and cefotaxime-plus-ciprofloxacin groups.

b Comparison of the ciprofloxacin and cefotaxime-plus-ciprofloxacin groups.

c Comparison of the cefotaxime-plus-minocycline and cefotaxime-plus-ciprofloxacin groups.

### *In vitro* effect of sub-inhibitory concentrations of antibiotics on RtxA1-mediated cytotoxicity and the transcription of *rtxA1*

**Figure 3:**
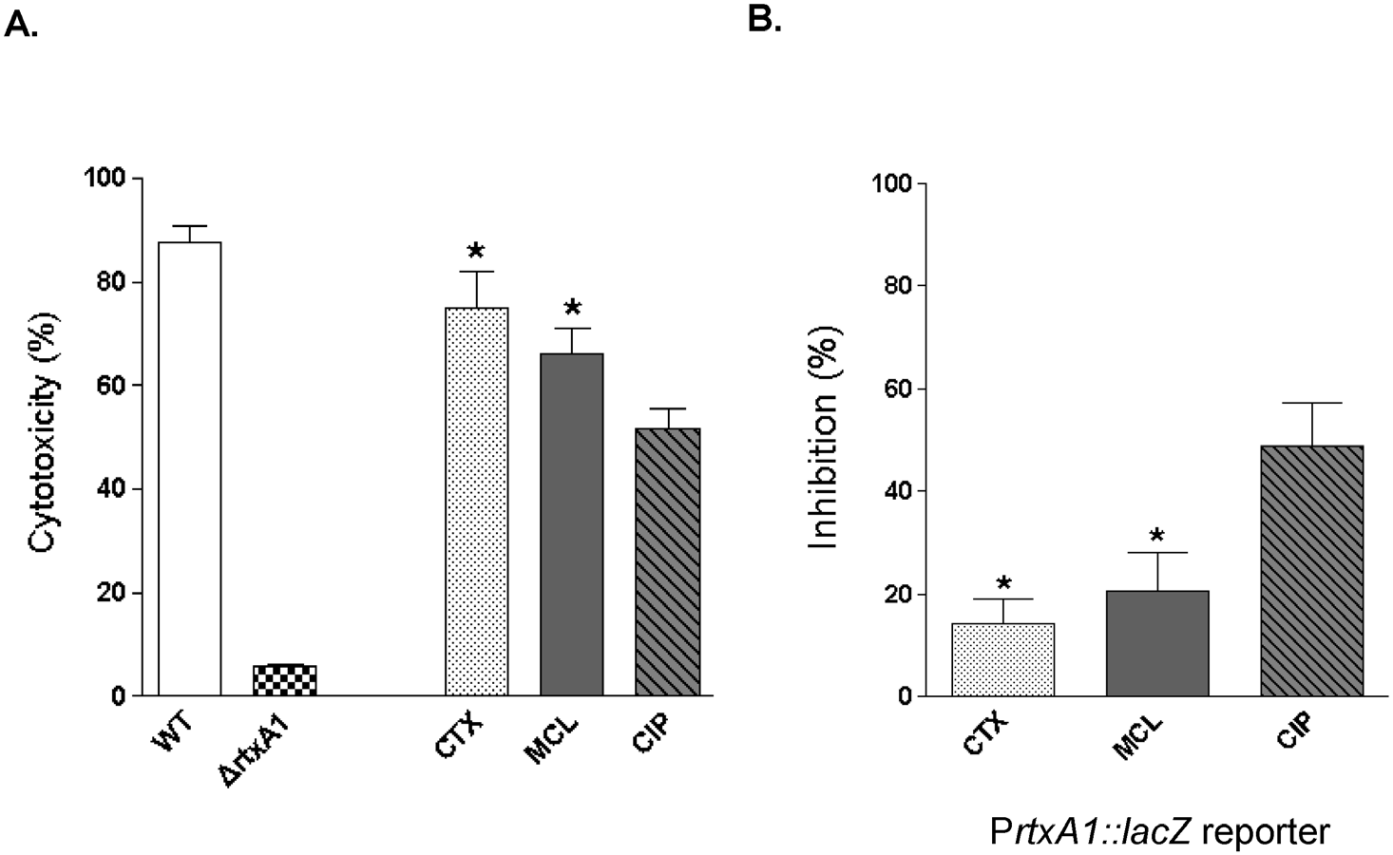
The effects of sub-inhibitory concentrations of antibiotics on *V. vulnificus* cytotoxicity and *rtxA1* transcription. A. Cytotoxicity assay. The impaired cytotoxicity of *ΔrtxA1* compared with that of the WT strain shows that cytotoxicity at 120 min is due principally to RtxA1. *V. vulnificus* cytotoxicity is inhibited more markedly by 1/4 MIC of ciprofloxacin than by 1/4 MICs of cefotaxime or minocycline (n = 12 per group). B. Transcriptional reporter assay. The transcription of *rtxA1* is more efficiently inhibited by 1/4 MIC of ciprofloxacin than by 1/4 MICs of cefotaxime or minocycline (n = 4 per group). WT, MO6-24/O; *ΔrtxA1*, CMM770 (MO6-24/O background with a deletion mutation in the *rtxA1* gene); CTX, cefotaxime; CIP, ciprofloxacin; MCL, minocycline. *P<0.05 compared to the values for ciprofloxacin (Student’s *t*-test).

The MICs of cefotaxime, minocycline, and ciprofloxacin for *V. vulnificus* MO6–24/O were 0.0625, 0.0625, and 0.03 mg/L, respectively. The impaired cytotoxicity of CMM770 (*ΔrtxA1*), as compared with MO6–24/O (WT), indicates that the cytotoxicity noted at 120 min was mainly due to RtxA1 (Fig. 3A). *V. vulnificus*-induced cytotoxicity was inhibited more effectively by 1/4 MIC of ciprofloxacin than by 1/4 MIC of cefotaxime or 1/4 MIC of minocycline (*P*<0.05 for each). Similarly, the transcription of *rtxA1* was inhibited more efficiently by 1/4 MIC of ciprofloxacin than by 1/4 MIC of cefotaxime or 1/4 MIC of minocycline (*P*<0.05 for each; Fig. 3B).

## Discussion

A previous *in* *vitro* study demonstrated a synergistic bactericidal effect of cefotaxime-plus-ciprofloxacin against *V. vulnificus* ATCC 27562 [22]. In the present study, we demonstrated that the combination of ciprofloxacin-plus-cefotaxime acts synergistically, and exhibits more potent bactericidal activity *in* *vitro* against *V. vulnificus* CMCP6 than either cefotaxime-plus-minocycline or ciprofloxacin alone. *In vitro* synergism for the combination of cefotaxime-plus-ciprofloxacin against enteric Gram-negative pathogens also has been reported for *E. coli* [19], *Serratia marcescens* [31], *Pseudomonas aeruginosa* [32], and *Salmonella enterica* serotypes Typhi and Paratyphi [20], [21], [33]. One possible mechanism underlying this synergism may be the interaction of quinolones with the outer membrane, with the quinolones acting as chelating agents, thereby increasing its permeability to β-lactam antibiotics [19].

We performed a survival analysis using 10^8^ cfu *V. vulnificus* as the initial inoculum, as a previous study showed *in* *vivo* synergism for cefotaxime and minocycline [14]. In that study, the mortality rates of cefotaxime-treated and cefotaxime-plus-minocycline-treated mice were 0% and 40%, respectively, similar to our results of 0% and 35%, respectively. However, the efficacy of quinolones was not directly compared with the other regimens using a high inoculum (10^8^ cfu), as a subsequent study using quinolones [13] was performed with a lower inoculum (1.5×10^7^ cfu) of *V. vulnificus.* Here, the survival rate was higher among mice treated with ciprofloxacin-based regimens than among mice treated with the cefotaxime-minocycline combination, although the *in* *vivo V. vulnificus* clearance rates for ciprofloxacin monotherapy and cefotaxime-plus-minocycline therapy were similar (*P* = 0.25, 0.32, 0.68, 0.84 in liver at 24 h, spleen at 24 h, liver at 48 h, spleen at 48 h).

To compare the efficacies of the treatment regimens on host survival and bacterial numbers in the organs, we infected mice with 1×10^7^ cfu *V. vulnificus*. Although all infected control mice died rapidly, all of the mice in the treatment groups survived, regardless of the regimen used, including cefotaxime monotherapy. Similar results were reported by Tang *et al*. [13], who compared the efficacies of fluoroquinolone and other agents using 1.5×10^7^ cfu *V. vulnificus*; they found that the survival rates of the mice were excellent at >80%, regardless of the administered regimen. Kim *et al*. [30] performed a survival analysis after antibiotic therapy using 10^8^ cfu *V. vulnificus* as the initial inoculum [34], based on the finding that none of the mice that were inoculated with 10^7^ cfu *V. vulnificus* and treated with antibiotics died, regardless of the regimen (personal communication). We considered it unfeasible to compare the survival rates for different antibiotic regimens when <10^8^ cfu/mL *V. vulnificus* were used as the initial inoculum, given that many samples would be needed to show a statistically significant difference if the survival rate was >80% for all regimens used. Therefore, we enumerated the bacteria in the organs of the inoculated and treated mice and found that cefotaxime-ciprofloxacin was the most effective regimen in terms of clearing *V. vulnificus*. These data provide further evidence for the superior efficacy of cefotaxime-plus-ciprofloxacin regimens in clearing *V. vulnificus in* *vivo*.

Sub-inhibitory concentrations of antibiotics interfere with the processes of host-parasite interactions, such as phagocytosis, adherence, and toxin production. For this reason, the anti-toxin effects of sub-inhibitory levels of antibiotics have been studied specifically in pathogens that cause rapidly fatal toxin-related diseases, such as necrotizing skin and soft tissue infections, in which anti-toxin efficacy is therapeutically important [35]–[38]. In our previous studies, we showed that RtxA1 is a major virulence factor of *V. vulnificus* [3], [7], and that deletion of *rtxA1* or its regulators decrease the cytotoxicity and increase the LD_50_ significantly in mice [6], [25], [28], [30], [39]. We also showed that RtxA1 can be suppressed by certain compounds [29], [40] and sub-inhibitory concentrations of chloramphenicol [25]. However, although RtxA1 is known as a major virulence element in *V. vulnificus*, and *V. vulnificus* causes rapidly fatal necrotizing skin and soft tissue infections, the effects of sub-inhibitory concentration of antibiotics that are frequently used in therapy on RtxA1 production have not been evaluated to date. In the present study, we showed that ciprofloxacin more effectively suppressed *rtxA1* transcription and protected host cells than either minocycline or cefotaxime. In a previous study, suppressing RtxA1 production *per se* without direct killing of bacteria was shown to reduce the mortality rate of mice infected with *V. vulnificus* [29]. The more potent inhibitory effect of ciprofloxacin on RtxA1-induced cytotoxicity may explain the improved survival of mice treated with ciprofloxacin-based regimens, compared with mice treated with regimens using cefotaxime or doxycycline.

Certain conclusions that we can draw from this study are limited, as we examined only the combination of ciprofloxacin-plus-cefotaxime. Further studies will be needed to evaluate the efficacy of combinations of ciprofloxacin or minocycline and other third-generation cephalosporins, including ceftazidime and cefrtriaxone.

In summary, we found that, at sub-inhibitory concentrations, ciprofloxacin is more effective at reducing *rtxA1* transcription and subsequent cytotoxicity than either minocycline or cefotaxime. Moreover, we demonstrated that the combination of ciprofloxacin and cefotaxime is more effective in clearing *V. vulnificus in* *vivo* than commonly used regimens, which suggests that this combination is a good candidate for the treatment of *V. vulnificus* sepsis in humans.
